# Comprehensive analysis of the secreted proteome of adult *Necator americanus* hookworms

**DOI:** 10.1371/journal.pntd.0008237

**Published:** 2020-05-26

**Authors:** Jayden Logan, Mark S. Pearson, Srikanth S. Manda, Young-Jun Choi, Matthew Field, Ramon M. Eichenberger, Jason Mulvenna, Shivashankar H. Nagaraj, Ricardo T. Fujiwara, Pedro Gazzinelli-Guimaraes, Lilian Bueno, Vitor Mati, Jeffrey M. Bethony, Makedonka Mitreva, Javier Sotillo, Alex Loukas

**Affiliations:** 1 Centre for Molecular Therapeutics, Australian Institute of Tropical Health and Medicine, James Cook University, Cairns, QLD, Australia; 2 Cancer Data Science Group, ProCan, Children's Medical Research Institute, Faculty of Medicine and Health, University of Sydney, Westmead, NSW, Australia; 3 LifeBytes India Pvt Ltd, Whitefield, Bangalore, India; 4 McDonnell Genome Institute, Washington University School of Medicine, St. Louis, Missouri, United States of America; 5 QIMR-Berghofer Medical Research Institute, Brisbane, QLD, Australia; 6 Institute of Health and Biomedical Innovation and Translational Research Institute, Queensland University of Technology, Brisbane, QLD, Australia; 7 Department of Parasitology, Biological Sciences Institute, Federal University of Minas Gerais, Belo Horizonte, Brazil; 8 Department of Health Sciences, Universidade Federal de Lavras, Lavras, Brazil; 9 Department of Microbiology, Immunology and Tropical Medicine, George Washington University, Washington DC, United States of America; 10 Centro Nacional de Microbiología, Instituto de Salud Carlos III, Majadahonda, Madrid, Spain; The University of Melbourne, AUSTRALIA

## Abstract

The human hookworm *Necator americanus* infects more than 400 million people worldwide, contributing substantially to the poverty in these regions. Adult stage *N*. *americanus* live in the small intestine of the human host where they inject excretory/secretory (ES) products into the mucosa. ES products have been characterized at the proteome level for a number of animal hookworm species, but until now, the difficulty in obtaining sufficient live *N*. *americanus* has been an obstacle in characterizing the secretome of this important human pathogen. Herein we describe the ES proteome of *N*. *americanus* and utilize this information along with RNA Seq data to conduct the first proteogenomic analysis of a parasitic helminth, significantly improving the available genome and thereby generating a robust description of the parasite secretome. The genome annotation resulted in a revised prediction of 3,425 fewer genes than initially reported, accompanied by a significant increase in the number of exons and introns, total gene length and the percentage of the genome covered by genes. Almost 200 ES proteins were identified by LC-MS/MS with SCP/TAPS proteins, ‘hypothetical’ proteins and proteases among the most abundant families. These proteins were compared to commonly used model species of human parasitic infections, including *Ancylostoma caninum*, *Nippostrongylus brasiliensis* and *Heligmosomoides polygyrus*. SCP/TAPS proteins are immunogenic in nematode infections, so we expressed four of those identified in this study in recombinant form and showed that they are all recognized to varying degrees by serum antibodies from hookworm-infected subjects from a disease-endemic area of Brazil. Our findings provide valuable information on important families of proteins with both known and unknown functions that could be instrumental in host-parasite interactions, including protein families that might be key for parasite survival in the onslaught of robust immune responses, as well as vaccine and diagnostic targets.

## Author summary

Hookworms infect hundreds of millions of people in tropical regions of the world. Adult worms reside in the small bowel where they feed on blood, causing iron-deficiency anemia when present in large numbers and contributing substantially to the poverty in these regions. Hookworms inject excretory/secretory (ES) products into the gut tissue when they feed, and while the protein constituents of ES products have been characterized for a number of animal hookworm species, difficulty in obtaining sufficient live human hookworms has thus far precluded characterization of the secreted proteome. Herein we describe the ES proteins of the major human hookworm, *Necator americanus*, and utilize this information to significantly improve the available genome sequence. Almost 200 ES proteins were identified and compared to the secreted proteomes of other parasitic roundworms to provide a molecular snapshot of the host-parasite interface. We produced recombinant forms of some of the identified proteins and showed that they are all recognized to varying degrees by antibodies from hookworm-infected subjects. Our work sheds light on important families of proteins that might be key for parasite survival in the human host, and presents a dataset that can now be mined in the search for vaccine, drug and diagnostic targets.

## Introduction

Hookworm infection is one of the most pertinent and life-limiting parasitic infections worldwide, affecting more than 400 million people in tropical regions of Asia, Africa and South America [1, 2]. Chronic infection with hookworms results in fatigue, abdominal pain, diarrhea, weight loss and anemia [3]. In children, it can cause growth retardation and impairments in cognitive development, and in pregnant women these infections lead to poor birth outcomes including low birth weight, increased perinatal morbidity and mortality [4, 5]. Moreover, hookworm infections result in 3.2 million disability-adjusted life years lost annually [6]. Hookworm infection therefore contributes significantly to widespread poverty in the majority of endemic regions [7].

The life cycle of the most widespread of the anthropophilic hookworms, *Necator americanus*, is direct, with no intermediate hosts involved. Eggs are passed out of the body in the feces and, under favorable conditions, hatch releasing first-stage (L1). Larvae undergo several molts to reach the infective third (L3) stage which can penetrate human skin [8]. Upon infection the parasite migrates through the circulatory system to the lungs, where it moves up the trachea and is eventually swallowed, thus commencing its final sojourn through the gastrointestinal tract to reside in the small intestine where mature adult worms can live for up to 10 years [8]. The adult stage of *N*. *americanus* produces macromolecules known as excretory/secretory (ES) products, which consist of a battery of proteins that have evolved to interact with human host tissues and facilitate parasitism [9, 10]. These ES products have the potential to not only be targeted as potential vaccine and diagnostic candidates, but also to shed light on how these parasites evade immune destruction [11–14].

Despite their potential biotechnological utility, only a limited number of *N*. *americanus* ES proteins have been described to date, and most of them have been identified as cDNAs based on their homology to proteins from more readily accessible hookworm species from animals such as *Ancylostoma caninum* [15]. In terms of vaccine antigens, a handful of *N*. *americanus* ES products including glutathione-S-transferases, aspartic proteases and sperm-coating proteins/Tpx-1/Ag5/PR-1/Sc7 (SCP/TAPS), have been identified at the cDNA level, and vaccine efficacy of recombinant proteins assessed in animal models and phase 1 clinical trials [13, 16, 17]. SCP/TAPS, also referred to as venom allergen-like or Activation-associated Secreted Proteins (ASPs) (Pfam accession number no. PF00188) have been reported from many helminths, but appear to be significantly expanded in the genomes and secreted proteomes of gut-resident clade IV and V parasitic nematodes, including the hookworms [11, 18–20].

Considerably little is known about the roles of *N*. *americanus* proteins in immunoregulation compared with many of the parasites that serve as animal models for human infections. Hsieh and colleagues reported an *N*. *americanus* protein(s) which selectively bound Natural Killer cells resulting in IL-2- and IL-12-dependent IFN-γ production, although the identity of the protein was not determined [21]. Calreticulin, a protein from the ES products of *N*. *americanus*, was identified as having an immunomodulatory role through inhibition of the hemolytic capacity of C1q, a human complement protein [22]. The relative paucity of functional information on *N*. *americanus* proteins can be attributed, at least in part, to the difficulty in obtaining parasite material and the absence until 2014 of the published *N*. *americanus* draft genome. Analysis of the 244 Mb draft genome and the predicted 19,151 genes [11], provided important information about the molecular mechanisms and pathways by which *N*. *americanus* interacts with its human host. In agreement with published transcriptomes of *N*. *americanus* and genomes/transcriptomes of other hookworm species, a select number of protein families were over-represented, including SCP/TAPS proteins and different mechanistic classes of proteases with various functions including hemoglobinolysis, and tissue penetration [23–28]. Of the >19,000 predicted genes reported in the draft *N*. *americanus* genome, 8,176 genes had no known InterPro domain. Additionally, more than half of the total proteins had either no blast homology to any gene from the NCBI database (10,771 proteins) or shared identity with a ‘hypothetical’ protein (3,043 proteins). These results highlight the importance of further annotation and refinement of the *N*. *americanus* genome [29].

In general, the practicality of genomic sequence data is dependent on the accuracy of gene annotation as well as the availability of functional, expression and localization information [30]. The high-throughput methods used when annotating a genome are prone to errors, therefore, to validate the predicted protein-coding genes, an analysis of the proteome is essential. Mass spectrometry provides useful data that can be used in a proteogenomic approach to improve genome annotation and identify novel peptides containing predicted protein sequences [31].

Herein we perform the first proteogenomic analysis of a parasitic helminth, while also significantly improving the genome annotation and comprehensively characterizing the ES proteome of adult *N*. *americanus*. Our findings provide valuable information on important families of proteins with both known and unknown functions that could be instrumental in host-parasite interactions, including protein families that might be key for parasite survival and protection of the host against excessive immunopathology. Moreover, we show that SCP/TAPS proteins are immunogenic in human hookworm infections, and with further effort could form the basis of a serodiagnostic test for hookworm infections.

## Methods

### Experimental Design and Statistical Rationale

A total of 69 fractionated samples were analysed, including two biological replicates for the ES (49 fractions from 2 biological replicates) and one biological replicate for the L3 somatic samples (20 fractions). Each biological replicate from the ES samples was fractionated by SDS-PAGE or Offgel to maximise sample separation and peptide discovery and analysed by mass spectrometry (25 from SDS fractions and 24 from Offgel fractions). L3 somatic samples were fractionated by SDS-PAGE. Adult somatic data was obtained from previous studies [11]. Different search engines were employed to increase the confidence of identified PSMs and maximize peptide identification [32], including Mascot, X!Tandem and Comet as described in section “Protein identification”. For peptide and protein identification, a False Discovery Rate (FDR) of 1% was applied.

### Ethics

Ethical approval for hamster animal experimentation to obtain *N*. *americanus* adult worms was obtained according to the Brazilian Guidelines on Animal Work, and approved by the local Animal Ethics Committee (CEUA) of the Federal University of Minas Gerais (Protocol# 51/2013). Ethical approval for human experimental infection with *N*. *americanus* and subsequent culturing of L3 was obtained from the James Cook University Human Research Ethics Committee (ID# H5936).

### Parasite material

Adult *N*. *americanus* were manually isolated from the intestines of experimentally infected golden hamsters (*Mesocricetus auratus*) upon euthanasia. For the isolation and purification of ES products, the worms were washed 3 times in phosphate-buffered saline (PBS) before being cultured overnight in a humidified incubator at 37°C, 5% CO_2_, in RPMI 1640 (100U/ml penicillin, 100 μg/ml streptomycin sulphate, 0.25 μg/ml amphotericin B). The supernatant was collected the following day and debris was removed by centrifuging the concentrated samples at 1,500 g for 3 minutes in a benchtop microfuge. ES products were concentrated with a 3 kDa cut-off Centricon filter membrane (Merck Millipore) and samples were stored at -80°C until use. For somatic adult extracts, data was used from Tang *et al* [11]. In brief, whole worms were ground under liquid nitrogen and solubilized using lysis buffer (1.0% (v/v) Triton X-100 in 40 mM Tris, 0.1% (w/v) SDS, pH 7.4). The extract was filtered through 20μm filter before fractionation.

For isolation of *N*. *americanus* L3, stool samples were collected from infected human volunteers and cultured as follows. Reverse osmosis water was added to the stool sample until a thick paste was formed. This paste was then distributed onto moistened Filter Paper (VWR, Standard Grade, 110 mm) in Petri dishes (Sarstedt, 150 mm) and placed in a 25°C incubator for 8 days. Following incubation, the edges of each plate were gently rinsed with RO water to obtain clean L3 preparations. Somatic extracts were prepared by adding 100 μl lysis buffer (3 M Urea, 0.2% SDS, 1% Triton X, 50 mM Tris-HCl) to approximately 6,000 larvae before repeated vortexing and sonication (4°C, probe sonicator, pulse setting) to digest the larvae. The extract was passed through a 0.45 μm filter (Millipore) before in-gel fractionation.

### SDS-PAGE fractionation and in-gel trypsin digestion

A total of 30 μg of *N*. *americanus* adult ES products was buffer exchanged into 50 mM NH_4_HCO_3_, freeze-dried and resuspended in Laemmli buffer. The sample was boiled at 95°C for 5 minutes and electrophoresed on a 12% SDS-PAGE gel for 40 minutes at 150V. The gel was stained with Coomassie Blue and 20 pieces (approximately 1 mm thick) were cut and placed into Eppendorf tubes. For the in-gel digestion, slices were de-stained and freeze-dried before incubating them at 65°C for 1 h in reduction buffer (20 mM dithiothreitol (DTT), Sigma, 50 mM NH_4_HCO_3_). Samples were then alkylated in 50 mM iodoacetamide (IAM, Sigma), 50 mM NH_4_HCO_3_ for 40 minutes at 37°C, washed three times with 25 mM NH_4_HCO_3_ and digested with 20 μg/ml of trypsin (Sigma) by incubating them for 16 h at 37°C with gentle agitation. Digestion was stopped and peptides were released from the slices by adding 0.1% TFA, 70% acetonitrile. This step was repeated 3 times with pooling of the corresponding supernatants to maximise peptide recovery for each sample. Finally, each sample was desalted with a ZipTip (Merck Millipore) and stored at -80°C until use.

### In-solution trypsin digestion and off-gel fractionation

A total of 70 μg of each *N*. *americanus* extract (L3 somatic and adult ES products) was buffer exchanged into 50 mM NH_4_HCO_3_ before adding DTT to 20 mM and incubating for 10 minutes at 65°C. Alkylation was carried out by adding IAM to 55 mM and incubating for 45 minutes at room temperature in the dark. Samples were digested with 2 μg of trypsin by incubating for 16 h at 37°C with gentle agitation. Following trypsin digestion, peptides were fractionated using a 3100 OFFGEL Fractionator (Agilent Technologies) according to the manufacturer’s protocol with a 24-well format as described previously [19]. In brief, Immobiline DryStrip pH 3–10 (24 cm) gel strips were rehydrated in rehydration buffer in the assembled loading frame. Digested peptides were diluted in dilution buffer to a volume of 3.6 ml and loaded equally across the 24 well cassette. The sample was run at a current of 50 μA until 50 kilovolt hours had elapsed. Upon completion of the fractionation, samples were collected, desalted with ZipTip and stored at -80°C until use.

### Mass spectrometry

The extracts were analyzed by LC-MS/MS on a Shimadzu Prominence Nano HPLC (Japan) coupled to a Triple Time of Flight (TOF) 5600+ mass spectrometer (SCIEX, Canada) equipped with a nano electrospray ion source. Fifteen (15) μl of each extract was injected onto a 50 mm x 300 μm C18 trap column (Agilent Technologies, Australia) at 60 μl/min. The samples were de-salted on the trap column for 6 minutes using 0.1% formic acid (aq) at 60 μl/min. The trap column was then placed in-line with the analytical nano HPLC column, a 150 mm x 100 μm 300SBC18, 3.5 μm (Agilent Technologies, Australia) for mass spectrometry analysis. For peptide elution and analysis the nano-HPLC pump was initially held at 2% solvent B for 6 minutes followed by a linear gradient of 2–40% solvent B over 80 minutes at 500 nl/minute flow rate and then a steeper gradient from 40% to 80% solvent B in 10 minutes was applied. Solvent B was held at 80% for 5 minutes for washing the column and returned to 2% solvent B for equilibration prior to the next sample injection. Solvent A consisted of 0.1% formic acid (aq) and solvent B contained 90/10 acetonitrile/ 0.1% formic acid (aq). The ionspray voltage was set to 2200V, declustering potential 100V, curtain gas flow 25, nebulizer gas 1 12 and interface heater at 150°C. The mass spectrometer acquired 250 ms full scan TOF-MS data followed by 20 by 250 ms full scan product ion data in an Information Dependent Acquisition mode. Full scan TOF-MS data was acquired over the mass range 300–1600 and for product ion ms/ms 80–1600. Ions observed in the TOF-MS scan exceeding a threshold of 150 counts and a charge state of +2 to +5 were set to trigger the acquisition of product ion, MS/MS spectra of the resultant 20 most intense ions. The data was acquired and processed using Analyst TF 1.6.1 (SCIEX, Canada).

### Proteogenomics

The mass spectrometry raw data was searched against the *N*. *americanus* protein database [11] using SequestHT algorithm in Proteome Discoverer 2.1 (Thermo Scientific, Bremen, Germany). Trypsin was used as the protease, allowing a maximum of two missed cleavages. Carbamidomethylation of cysteine was specified as a fixed modification, and oxidation of methionine was included as variable modifications. The minimum peptide length was specified as 6 amino acids. The mass error of parent ions was set to 10 ppm, and for fragment ions it was set to 0.05 Da. Protein inference was based on the rule of parsimony and required one or more unique peptides. FDR of 1% at peptide and protein levels was applied. The unassigned spectra from the protein database search were further searched against the six-frame translated genome database of *N*. *americanus* [11]. The genome sequences were downloaded from WormBase ParaSite release 9 (http://parasite.wormbase.org/Necator_americanus_prjna72135/Info/Index) in FASTA format and translated in all six frames using in-house python scripts. All the sequences greater than 10 amino acids between any two stop codons were added to the database. The search was again performed using SequestHT with precursor mass tolerance of 50 ppm and fragment ion tolerance of 1 Da. Carbamidomethylation of cysteine was specified as a fixed modification, and oxidation of methionine was included as variable modifications. Results were obtained at 1% FDR for both protein and peptide level.

The identified peptides in the search against the six-frame translated genome were mapped back to the protein database using standalone BLAST. Any sequences that mapped 100% to the protein database were discarded. The filtered peptides were mapped to the *N*. *americanus* genome using the standalone tblastn program [11]. Peptide identifications that unambiguously mapped to a single region in the genome, also known as Genome Search Specific Peptides (GSSPs), were considered to perform proteogenomics-based annotation of novel coding regions. The known gene annotation GFF3 was downloaded from WormBase ParaSite for *N*. *americanus* [11]. Using in-house scripts, we categorized the GSSPs into various categories: intergenic, intronic, exon-extension, alternative frame, N-terminal extensions or repeat regions with respect to the known regions from GFF3. Additionally, for all the intergenic peptides, we determined if there was a potential open reading frame (ORF) within the stretch of amino acids. Each of the spectra was further manually validated. All newly identified ORFs were blasted against a custom database containing the proteomes of *A*. *caninum*, *A*. *ceylaninum* and *A*. *duodenale* in order to annotate them. Interpro IDs were used to identify functional domains and SMART (http://smart.embl-heidelberg.de/) domain prediction software was used independently to determine which sequences contained a functional domain.

### Genome annotation

The genome annotation from *N*. *americanus* [11] was updated using the MAKER pipeline v2.31.8 [33]. The genome assembly (GenBank assembly accession: GCA_000507365.1) was softmasked for repetitive elements with RepeatMasker v4.0.6 using a species-specific repeat library created by RepeatModeler v1.0.8, RepBase repeat libraries [34] and a list of known transposable elements provided by MAKER [33]. From the NCBI Sequence Read Archive, *N*. *americanus* RNA-Seq data [11] (Adult: SRR609895, SRR831085, SRR892200; L3: SRR609894, SRR831091, SRR89220) were obtained. After adapter and quality trimming using Trimmomatic v0.36 [35], RNA-Seq reads were aligned to the genome using HISAT2 v2.0.5 [36] with the—dta option and subsequently assembled using StringTie v1.2.4 [37]. The resulting alignment information and transcript assembly were used by BRAKER [38] and MAKER pipelines, respectively, as extrinsic evidence data. In addition, protein sequences from SwissProt UniRef100 [39] and WormBase ParaSite WS258 [40] (*Ancylostoma ceylanicum* PRJNA231479, *Brugia malayi* PRJNA10729, *Caenorhabditis elegans* PRJNA13758, *Onchocerca volvulus* PRJEB513, *Pristionchus pacificus* PRJNA12644, *Trichinella spiralis* PRJNA12603 and *Strongyloides ratti* PRJEB125) were provided to MAKER as protein homology evidence. Following the developer’s recommendation [41], the protein-coding gene models of Tang *et al*. [11] were passed to MAKER as pred_gff to update the models by adding new 3’ and 5’ exons, additional UTRs, and merging split models. This method, however, cannot change internal exons nor create new annotations where evidence suggests a gene but no corresponding model is previously present. To address this shortcoming, additional *ab initio* gene predictions were generated using BRAKER v2.0.1 [38] and passed to MAKER so that the intron-exon model that best matched the evidence could be included in the final annotation set. Within the BRAKER pipeline, the gene prediction tools GeneMark [42] and AUGUSTUS [43] were trained utilizing the *N*. *americanus* RNA-Seq alignment and protein homology information from *C*. *elegans*. The GFFPs identified in the present study were used to confirm the validity of proposed annotation changes and resolve competing gene predictions through manual curation. Gene models with no evidence support were not included in the final annotation build to reduce false positives in the existing annotations. However, *ab initio* gene predictions that encoded Pfam domains as detected by InterProScan v5.19 [44] were rescued to enhance overall accuracy by balancing sensitivity and specificity [33, 45]. Our approach, while being conservative, relied on the quality and completeness of previously published genomes, and may have missed some of species-specific gene models. The completeness of the annotated gene set was assessed using BUSCO v3.0 with Eukaryota-specific single copy orthologs (OrthoDB v9) [46].

### Protein identification

Mascot version 2.5 (Matrix Science), X!Tandem (The Global Proteome Machine Organisation) version Jackhammer and Comet v2014.02 rev.2 were used to analyze data from the mass spectrometer. Searches were carried out against a database comprised of either the updated genome annotation provided with this study (15,728 sequences), or the *N*. *americanus* genome [11] (19,153 sequences), both appended to the common repository of adventitious proteins (cRAP; http://www.thegpm.org/crap/; 116 sequences) database (to detect potential contamination) and their corresponding decoy sequences (total of 38,538 sequences when using the *N*. *americanus* previous published genome version [11] and 31,688 when using the updated genome annotation). The following parameters were used: enzyme, trypsin; variable modifications, oxidation of methionine, carbamidomethylation of cysteine, deamidation of asparagine and glutamine; maximum missed cleavages, 2; precursor ion mass tolerance, 50 ppm; fragment ion tolerance 0.1 Da; charge states, 2+, 3+ 4+. A FDR of 0.1% was applied, and a filter of greater than 2 significant unique sequences was used to further improve the robustness of data. The mass spectrometry data have been deposited in the ProteomeXchange Consortium via the PRIDE partner repository with the dataset identifier PXD010669. A False Discovery Rate (FDR) of 1% at peptide and protein levels was applied in all searches.

### Bioinformatic Analysis of Proteomic Sequence Data

Gene ontology (GO) annotations were assigned using the program Blast2GO and Pfam analysis was performed using HMMER [47]. Pfam domains were detected at the P<0.001 threshold for the HMMER software. Putative signal peptides were predicted with SignalP v4.1 and transmembrane domains with TMHMM v2.0 [48, 49]. REViGO, an online tool, was used to summarise and plot GO terms [50]. UpSetR was used to group proteins based on whether they had a GO term with one, two or all of the GO categories (biological process, molecular function or cellular process) [51].

### Similarity analysis

A similarity analysis was carried out based on the Parkinson and Blaxter method using an in-house script [26, 52]. Given the difficulty of working with *N*. *americanus* specifically (in terms of accessibility to samples and establishment of life cycle in other hosts), other hookworms are frequently used to model this human parasite. Data from the secreted proteome of adult *N*. *americanus* (described herein) was compared to the published secreted proteomes from related adult nematode species including *H*. *polygyrus*, *A*. *caninum* and *Nippostrongylus brasiliensis* [15, 19, 53].

### Protein family similarity visualization

*H*. *polygyrus*, *A*. *caninum* and *N*. *brasiliensis* SCP/TAPS and protease protein sequences were obtained from their respective published secreted proteomes [15, 19, 53]. These sequences were aligned with adult *N*. *americanus* homologous protein sequences using BLAST. Significant sequence alignments were visualized using Circos [54].

### Phylogenetic analyses

SCP/TAPS proteins were identified from published secretomes of 6 species of parasitic helminths including *A*. *caninum*, *Ascaris suum*, *H*. *polygyrus*, *N*. *brasiliensis*, *Trichuris muris* and *Toxocara canis*. SCP/TAPS proteins were identified from *N*. *americanus* ES products using the proteome data generated in this study. Sequences were sorted into single and double SCP/TAPS domain proteins for individual phylogenetic analysis due to distinct differences described previously [55]. A list of the proteins and their respective sequences used for this analysis can be found in S1 Table. A multiple sequence alignment was carried out using the alignment program MUSCLE. Outliers with poor alignment (long unaligned regions) were detected and filtered out using ODseek. PhyML, a phylogeny software, was used for a maximum-likelihood phylogenetic analyses with bootstrapping of SCP/TAPS amino acid sequences. The tree was visualized with The Interactive Tree of Life (iTOF) online phylogeny tool (https://itol.embl.de/) [56].

### Recombinant protein expression and purification

We selected four SCP/TAPS protein family members representing double- (NAME_01818; NAME_01068; NAME_1070) and a single-domain (NAME_10402) protein from distinct phylogenetic clades for immunogenicity studies. We had proteomic evidence for secretion of the three double-domain proteins but there was no evidence of secretion of NAME_10402 in adult worm ES products, despite its upregulation at the mRNA level in the adult worm [11]. Complete open reading frames (without their signal peptides) were codon optimised for expression in the yeast *Pichia pastoris*, commercially synthesized and cloned into the pPICZαA expression vector (ThermoFisher) by Integrated DNA Technologies (Singapore). Sequences were cloned into the *EcoR*I and *Xba*I sites of pPICZαA such that they were in frame with the vector’s alpha-factor secretion signal (to facilitate secretion of the expressed protein into culture media) and C-terminal 6xHis tag to facilitate purification by Immobilised Metal Affinity Chromatography. Recombinant plasmids (40 μg) were linearized by digestion with *Sac*I, purified by phenol:chloroform extraction and ethanol precipitation and resuspended in 20 μl of H_2_O. Linearized vector was transformed into electrocompetent *P*. *pastoris* X-33 cells by electroporation (2 ms in 2 mm cuvettes using a BioRad Gene Pulser Xcell—2 kV, 25 μF capacitance, 200 Ohms resistance, square wave electroporation), plated onto yeast extract peptone dextrose agar plates supplemented with 2,000 μg/mL zeocin, covered in foil and incubated overnight for 3 days at 28°C. Integration of the expression cassette into the yeast chromosome was confirmed by colony PCR. Ten positive colonies were each seeded into 2 mL of buffered glycerol-complex (BMGY) media in 50 mL tubes, incubated at 30°C overnight with shaking at 250 rpm and the resultant cells centrifuged (2,500 *g* for 5 mins at 10°C), resuspended in 3 mL of buffered minimal methanol-complex medium (BMMY) to induce expression and similarly incubated for 96 hours. Cultures were supplemented with 0.5% methanol every 24 h to maintain promoter activity and 20 μL samples of culture media were taken every day and analysed for protein expression by Western blotting with an anti-His-HRP monoclonal antibody (ThermoFisher) using standard methods. A 10 μL sample of the highest expressing culture of each SCP/TAPS domain-containing protein was used to inoculate 25 mL of BMGY in a 250 mL conical flask, which was cultured overnight with shaking (250 rpm) at 30°C. The 25 mL culture was then used to inoculate 250 mL of BMGY in a 2.5 L conical flask, which was cultured overnight with shaking (250 rpm) at 30°C. To induce protein expression, the cells were pelleted at 2,500 *g* for 30 mins at 10°C, and then resuspended in 2 L of BMMY and split between four 2.5 L conical flasks, which were incubated with shaking (250 rpm) at 30°C for 96 hours. Methanol was added to a final concentration of 0.5% (2.5 mL/flask) every 24 hours to maintain promoter activity. Cells were pelleted at 2,500 *g* for 30 mins at 10°C. The supernatant was decanted, filtered through a 0.45 μm filter and applied to a pre-packed 5.0 mL His-Trap Excel IMAC column (GE Healthcare), that was charged with 100 mM nickel sulfate and equilibrated with 10 column volumes of binding buffer (50 mM Na_2_HPO_4_/NaH_2_PO_4_, pH 8, 300 mM NaCl, 10 mM imidazole), at a flow rate of 1.0 mL/min. Bound proteins were gradient-purified by washing and eluting with binding buffer containing an increasing concentration of imidazole (40–500 mM). Fractions taken throughout the purification process were analyzed by 12% SDS-PAGE and eluate fractions that contained protein were pooled, the concentration adjusted to 1.0 mg/mL using a 3 kDa cut-off Centricon filter (Merck Millipore) and verified using gel densitometry in comparison with BSA standards.

### Enzyme-Linked Immunosorbent Assay using human sera

The diagnostic potential of each SCP/TAPS protein was assessed by indirect IgG ELISA using a cohort of hookworm-positive (grouped according to World Health Organisation stratification: heavy infection—≥4,000 eggs per gram of faeces (epg), n = 10; moderate infection—2,000–3,999 epg, n = 11; or light infection—≤1,999 epg, n = 44), hookworm-negative (n = 7) and hookworm-negative/*Ascaris*-positive (n = 12) serum samples from a hookworm-endemic region in Minas Gerais, Brazil. In 2005, 1,494 individuals between the ages 4 and 66 years (inclusive) were enrolled (with informed consent) into a cross-sectional study in an *N*. *americanus*-endemic area of Northeastern Minas Gerais state in Brazil, using protocols approved by the George Washington University Institutional Review Board (117040 and 060605), the Ethics Committee of Instituto René Rachou and the National Ethics Committee of Brazil (CONEP; protocol numbers 04/2008 and 12/2006). Venous blood (15 mL) was collected from individuals determined to be positive for *N*. *americanus*. Microlon high-binding 96-well microtiter ELISA plates (Greiner) were incubated overnight at 4°C with each antigen (2 μg/ml in 0.1 M Na_2_CO_3_/NaHCO_3_, pH 9.6). Somatic extract of *N*. *americanus* L3 was similarly used as a positive control antigen. After washing with PBST (PBS + 0.05% Tween), the plates were blocked for 2 hours at RT with 100 μl of PBST/5% BSA. One hundred microliters of sera (1:200 in PBST/1% BSA) were added to the wells and incubated overnight at 4°C, then the plates were washed with PBST and 100 μl of goat anti-human IgG-HRP (Sigma) (1:5000 in PBST) was added to the plates. Plates were incubated for 2 hours at RT and then washed with PBST. Plates were developed by adding 100 μl of 3,3’,5,5’-tetramethylbenzidine and incubating for 30 minutes at RT in the dark. One hundred microliters of 1M HCl was added to stop the colorimetric reaction, which was read at a wavelength of 450 nm on a Polarstar Omega microplate reader (BMG Labtech). Assays were performed in triplicate and blank-corrected values were plotted using Graphpad Prism 7. The same software was also used to generate single and multi-antigen Receiver-Operating Characteristic (ROC) curves. Reactivity cutoffs were determined as the average plus 1SD of the values of the hookworm-negative group and the frequency of recognition of each antigen was determined using this cutoff.

## Results

### Proteogenomic analysis and genome annotation

The mass spectrometry proteomics data have been deposited to the ProteomeXchange Consortium via the PRIDE partner repository with the dataset identifier PXD010669. To access the data, please use the below login details.

Username: reviewer49665@ebi.ac.uk

Password: fybW0mgJ

Proteomes from two life stages (infective L3 somatic extract and adult ES products) of *N*. *americanus* were analysed by mass spectrometry in a 5600 ABSciex Triple Tof to perform a proteogenomic analysis of the hookworm genome. We identified 198 adult ES proteins and 590 L3 somatic proteins. In an earlier study we identified 458 proteins from a somatic extract of adult *N*. *americanus* [11], and those sequences were used herein to facilitate the annotation process. After excluding peptides that mapped accurately to the existing protein database entry, a total of 218 novel peptides were identified that did not match any protein sequences of *N*. *americanus* contained in the annotations of Tang *et al* [11]. Of these 218 novel peptides, 83 were found exclusively in adult somatic extracts, 50 exclusively in L3, and 67 exclusively in adult ES, while 10 peptides were found in both the adult somatic extract and ES products, and 8 were found in both adult and larval somatic extracts. No common peptides were identified from adult ES products and larval somatic extract.

**Fig 1:**
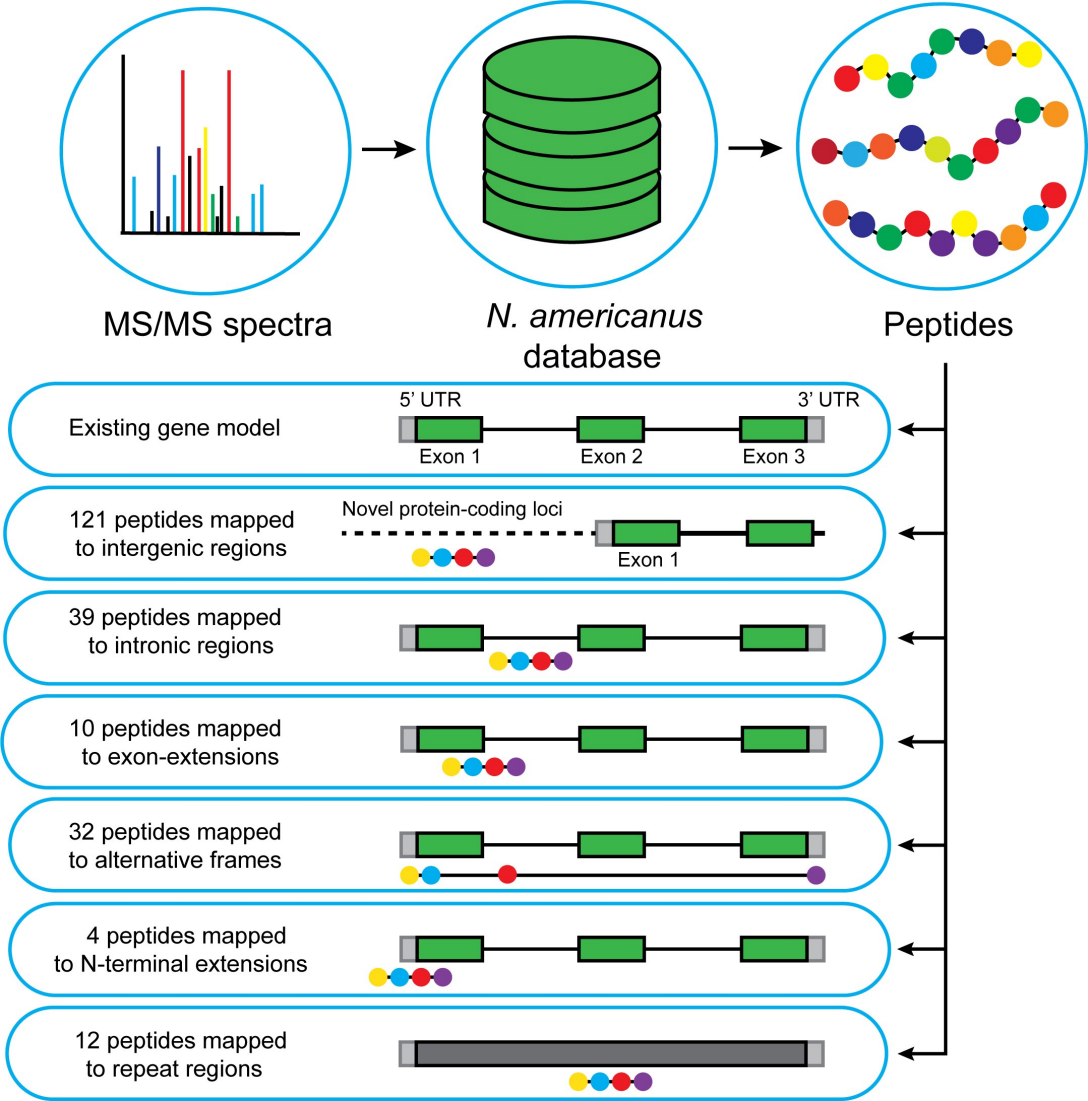
Depiction of the proteogenomic process as well as the types and numbers of peptide corrections identified.

Newly identified peptides could be grouped into the following six categories: (1) peptides mapping to intergenic regions; (2) peptides mapping to introns; (3) peptides mapping to alternative reading frames; (4) peptides extending gene boundaries (exon extensions); (5) peptides mapping to N-terminal extensions; and (6) peptides mapping to repeat regions (not gene) regions (Fig 1). Of the identified peptides, more than half belonged to group 1, 18% to group 2, 15% to group 3 and a small number to groups 4, 5 and 6 (Fig 1). The translated protein sequences with their respective peptides, six-frame translated genome ID and ORF length are provided in S2 Table. Group 1 peptides were further analyzed for an ORF including whether or not they contained a methionine; see S3 Table. From this data, one-third of group 1 peptides were found to overlap with a viable ORF, with the shortest having just 21 amino acids and the longest 381 amino acids. In addition, in order to further annotate these newly identified ORFs, we blasted the sequences against a custom database containing the proteomes of *A*. *caninum*, *A*. *ceylaninum* and *A*. *duodenale*. A total of 54 proteins were significantly similar to proteins from other ancylostomatids, while 7 had no known homologs (S4 Table). Furthermore, only 41 proteins contained a valid Pfam domain (S5 Table).

These results highlighted the need for improving the previously published gene models, so we updated the genome annotation of *N*. *americanus* using a new RNA-Seq based gene calling pipeline as outlined in the Materials and Methods (Table 1). The total number of predicted *N*. *americanus* genes decreased by 3,425, with substantial increases in both the number of exons and introns. The total coding sequence (CDS) length increased by 2.22 Mb with the mean gene length (including introns and UTRs) nearly doubling from 4.3 kb to 8.1 kb. Furthermore, the percentage of the genome covered by genes increased by 18.3%, and the percentage of detected BUSCOs among the predicted genes increased from 95.7% to 97.4% (with 31% reduction in the number of fragmented BUSCOs). While these improvements are attributable in most part to the use of more sophisticated genome annotation methods utilizing RNA-Seq data and the inclusion of more extensive, up-to-date homologous protein databases, the peptide sequences generated in this study contributed directly to the refinement of 14 gene models. The newly annotated, improved gene models were used in subsequent proteomic analysis of ES products, and are publicly available on Nematode.net [57, 58].

**Table 1:** Comparison of original and updated genome annotations.

	Original annotation	Updated annotation
Number of genes	19,153	15,728
Number of exons	122,849	148,780
Number of introns	103,696	133,052
Number of CDS	19,153	15,728
Overlapping genes	395	2,424
Contained genes	2	386
Total gene length (bp)	82,090,364	126,651,725
Total exon length (bp)	15,470,227	24,553,753
Total intron length (bp)	66,827,529	102,364,076
Total CDS length (bp)	15,461,420	17,683,227
Mean gene length (bp)	4,286	8,053
Mean exon length (bp)	126	165
Mean intron length (bp)	644	769
Mean CDS length (bp)	807	1,124
% of genome covered by genes	33.6	51.9
% of genome covered by CDS	6.3	7.2
Mean exons per mRNA	6.4	9.5
Mean introns per mRNA	5.4	8.5
Complete BUSCOs	82.84%	88.45%
Fragmented BUSCOs	12.87%	8.91%
Missing BUSCOs	4.29%	2.64%

### Analysis of the ES products from *N*. *americanus* adult worms

**Fig 2:**
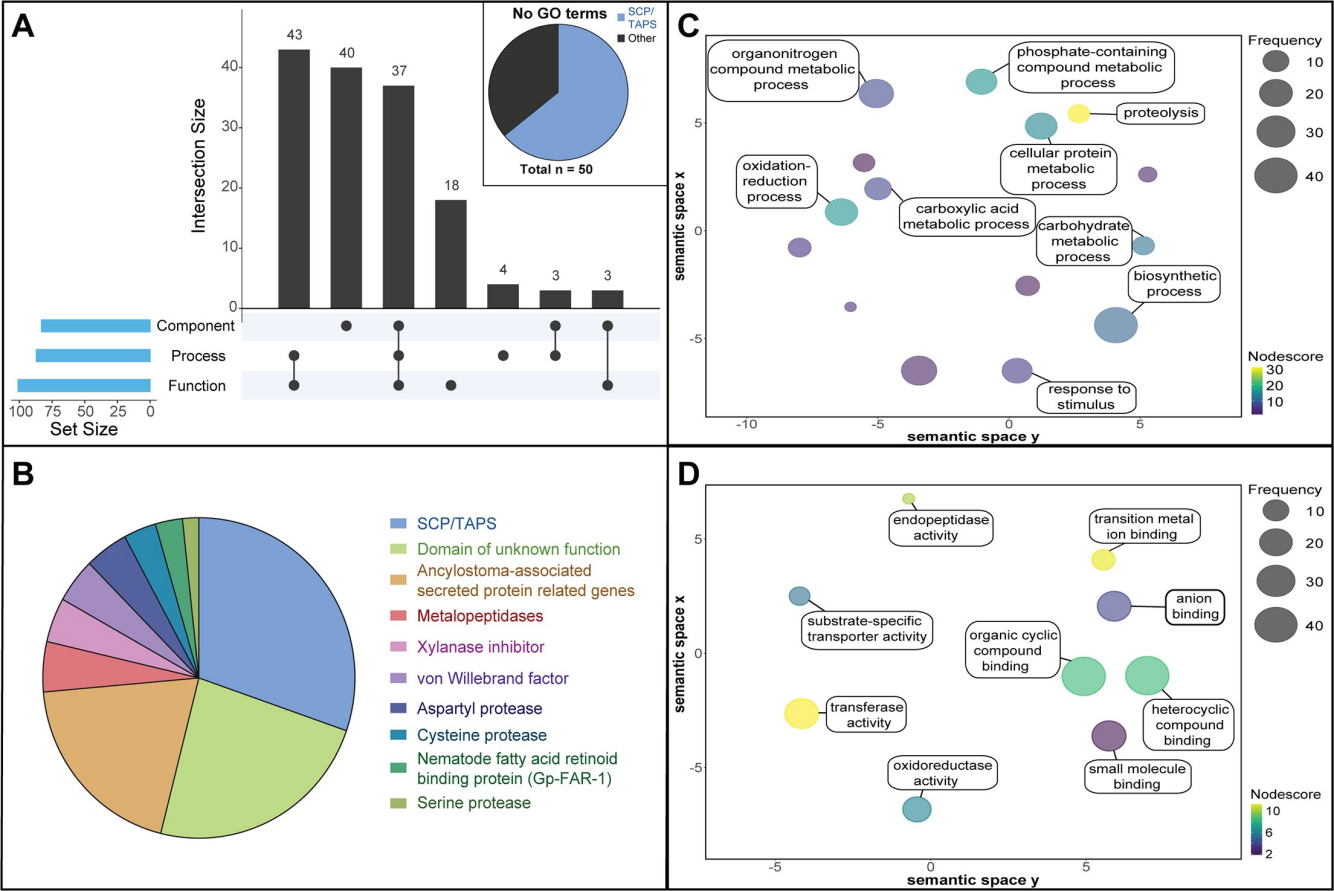
**(A)** UpSetR plot displaying the number of each gene ontology (GO) term categories (biological process, molecular function and/or cellular component) available (Blast2GO) for adult *Necator americanus* excretory/secretory (ES) proteins. Proteins with no available GO terms are broken down in a pie chart into ‘SCP/TAPS’ proteins ‘other’. **(B)** Top 10 most abundant protein families in the ES products of adult *N*. *americanus*. **(C)** Biological processes of adult *N*. *americanus* ES proteins ranked by nodescore (Blast2GO) and plotted using REViGO. Semantically similar GO terms plot close together, increasing heatmap score signifies increasing nodescore from Blast2GO, while circle size denotes the frequency of the GO term from the underlying database. **(D)** Molecular functions of adult *N*. *americanus* ES proteins ranked by nodescore (Blast2GO) plotted using REViGO.

A comprehensive analysis of the ES products from adult worms was carried out using in-gel and off-gel fractionation and the tryptic peptides were analyzed using LC-MS/MS. Mascot, X!Tandem and Comet searches were carried out against a database including the predicted proteins from the annotated *N*. *americanus* genome available in this study and the cRAP sequences available at http://www.thegpm.org/crap/. Identified contaminant and host proteins (*M*. *auratus)* are detailed in S6 Table. A total of 186 and 141 proteins were identified using Mascot and X!Tandem/Comet, respectively. The two search methods combined found a total of 198 proteins, S7 Table). These 198 proteins were obtained using the updated genome annotation generated as part of this study. In comparison, using the first version of the annotated genome sequence we identified 203 proteins using the same analytical software [11]. Using the exponential modified protein abundance index (emPAI) and the newly annotated genome, the most abundant proteins in the ES products of *N*. *americanus* adult worms were ranked, and the top 10 are shown in Table 2. The conserved Pfam domains of the 198 ES proteins identified were analyzed. The most abundant protein family in the ES products was the SCP/TAPS family with 54/198 proteins containing a single or double cysteine-rich secretory protein family (CAP) domain (PF00188) (Fig 2B). The top 10 most abundant protein families are displayed in Fig 2B. Many of these proteins were described by Blast2GO as ‘SCP-partial’ or ‘SCP-like’, but for a more standardized annotation in our analysis we have grouped them all as ‘SCP/TAPS’. The second most frequently represented group (with 42 proteins) were proteins with one or more domains of unknown function (DUF). Other abundant families included Ancylostoma secreted protein related (ASPR) proteins and metalloproteases with 35 and 9 of each identified respectively (S8 Table). Despite some published reports classifying ASPRs as SCP/TAPS proteins, they are a diverse set of secreted cysteine rich proteins based on Pfam annotation and therefore we have grouped them separately [28]. Of the 198 identified ES proteins, 48% contained a predicted signal peptide. Supporting the accuracy of the new gene model, 96 of the identified adult ES proteins were predicted to contain a signal peptide compared to just 75 using the previous genome annotation.

**Table 2:** Top 10 most abundant proteins in the excretory/secretory proteins of *N*. *americanus*.

	Blast2GO Description	MASCOT score		iProphet probabilty		NP				emPAI		
Accession		Ingel	Offgel	Ingel	Offgel	Ingel	Offgel	SP	TD	Ingel	Offgel	Domain
NAME_05596	Hypothetical protein	19496	15287	1	1	11	11	N	0	867.19	308.05	No conserved domains
NAME_07794	Hypothetical protein	21210	39596	1	1	18	22	Y	0	47.88	199.86	Single SCP
NAME_11917	SCP	35077	43195	1	1	18	31	N	0	11.87	68.4	Double SCP
NAME_13724	Hypothetical protein	7701	23099	1	1	5	7	N	0	7.06	64.55	No conserved domains
NAME_15197	SCP	9446	12866	1	1	6	7	N	0	9.55	50.11	No conserved domains
NAME_14329	SCP	10911	15142	1	1	10	15	N	0	14.34	43.25	Single SCP
NAME_11146	SCP	21158	29296	1	1	24	27	Y	0	17.03	22.85	Double SCP
NAME_10941	Hypothetical protein	983	3870	1	1	3	8	Y	0	2.6	33.23	Single SCP
NAME_09098	SCP	6908	47379	1	1	13	25	Y	0	3.27	31.87	Double SCP
NAME_05595	SCP-like protein, partial	0	23369	0	1	0	10	Y	0	0	26.61	Single SCP

In total, 30 GO terms were identified (following the removal of parent child redundancy) belonging to one of the three GO database categories: biological processes, molecular function or cellular component (Fig 2A, for raw data see S9 Table). Blast2GO returned biological process GO terms for 87/198 proteins, molecular function GO terms for 99/198 proteins and cellular component GO terms for 83/198 proteins (Fig 2A). The most prominent biological process term was proteolysis (Fig 2D), with 15% (29 proteins) of total ES products being involved in a proteolytic process. The most prominent single molecular function term was “hydrolase activity” followed by “peptidase activity” and “metal ion binding” (Fig 2C). Interestingly, 50/198 proteins did not return any GO terms (Fig 2A). Of these, SCP/TAPS proteins made up 64% with no known biological process, molecular function, or cellular component. This highlights a significant knowledge gap surrounding SCP/TAPS proteins produced by helminths.

### Similarity analysis of the ES products from different gastrointestinal nematode species

**Fig 3:**
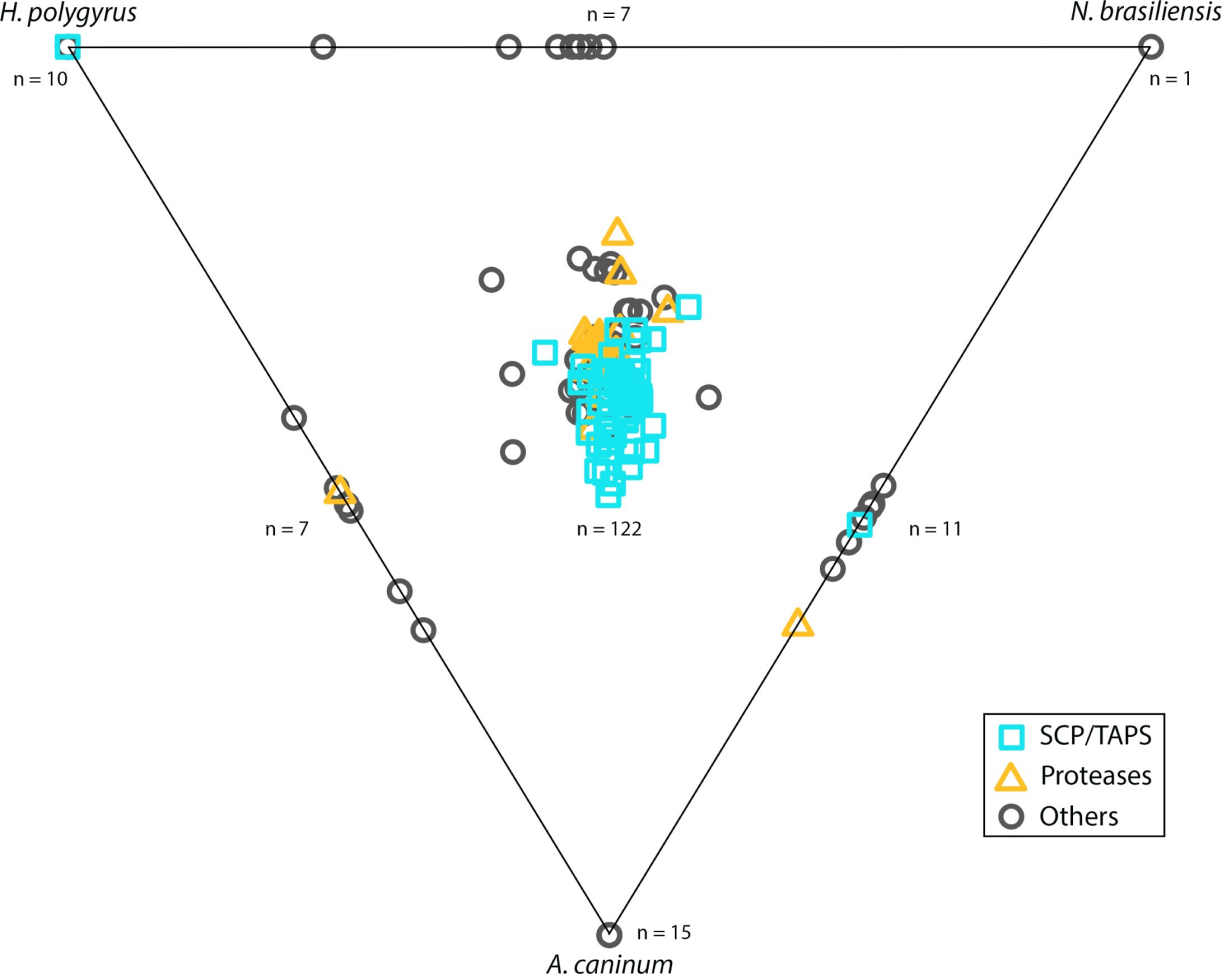
Excretory/secretory (ES) products from *Ancylostoma caninum*, *Heligmosomoides polygyrus*, *and Nippostrongylus brasiliensis* were compared with the ES proteome of *Necator americanus* and displayed in a Simitri plot. SCP/TAPS are represented by a turquoise triangle, proteases by an orange square and any other protein by a grey circle. Points on the diagram triangle represent sequences which only had similarity to the labelled species. Points along the edges of the triangle are sequences which had similarity to two of the three species (given at the respective ends of the edge). Any sequence in the middle area of the triangle represents a sequence with similarity to all three compared species.

A similarity analysis of ES proteomic data from *N*. *americanus* and three of the most commonly used animal models for human hookworms, *A*. *caninum*, *H*. *polygyrus* and *N*. *brasiliensis* was carried out (Fig 3). A total of 15, 10 and 1 *N*. *americanus* ES proteins had unique homology to ES proteins from *A*. *caninum*, *H*. *polygyrus* and *N*. *brasiliensis* respectively. This included one SCP/TAPS protein (NAME_13724) which was similar only to an *H*. *polygyrus* protein, while 3 of the proteins were similar only to *A*. *caninum* ES proteins with domains of unknown function (NAME_07734, NAME_09181, NAME_09182). Seven proteins shared homology with only *A*. *caninum* and *H*. *polygyrus* proteins, 11 shared homology with only *A*. *caninum* and *N*. *brasiliensis* proteins and 7 shared homology with only *H*. *polygyrus* and *N*. *brasiliensis* proteins. One hundred and twenty-two *N*. *americanus* proteins shared different degrees of homology with proteins from *A*. *caninum*, *H*. *polygyrus* and *N*. *brasiliensis*.

From the 54 SCP/TAPS proteins found in *N*. *americanus* ES products, one (NAME_13724) shared homology with a protein found in only *H*. *polygyrus*, while another SCP/TAPS protein (NAME_15177) shared homology with proteins from both *A*. *caninum* and *N*. *brasiliensis*. Fifty-one (51) of 54 SCP/TAPS proteins were similar to all compared species, leaving a single SCP/TAPS protein (NAME_11218) which did not have homology to any SCP/TAPS protein from the compared species. Twenty-five *N*. *americanus* ES proteins did not have homology to any proteins in the secretome of *A*. *caninum*, *H*. *polygyrus* or *N*. *brasiliensis*. Notable proteins among these 25 were NAME_01848 (aspartyl protease) and NAME_05081 (zinc metalloprotease).

Of the 26 proteases in the *N*. *americanus* ES products, 22 had homologs in all compared species, while one serine protease (NAME_06735) only had homologs in *H*. *polygyrus* and *A*. *caninum* and one metalloprotease (NAME_00535, peptidase family M1) only had homologs in the ES products of *A*. *caninum*, and *N*. *brasiliensis*. One zinc metalloprotease (NAME_05081) and one serine protease (NAME_01250) were only found in the ES products of *N*. *americanus* and therefore did not have any similarity to ES products from the other species.

### Homology analysis of SCP/TAPS and proteases in the ES products of *N*. *americanus*

**Fig 4:**
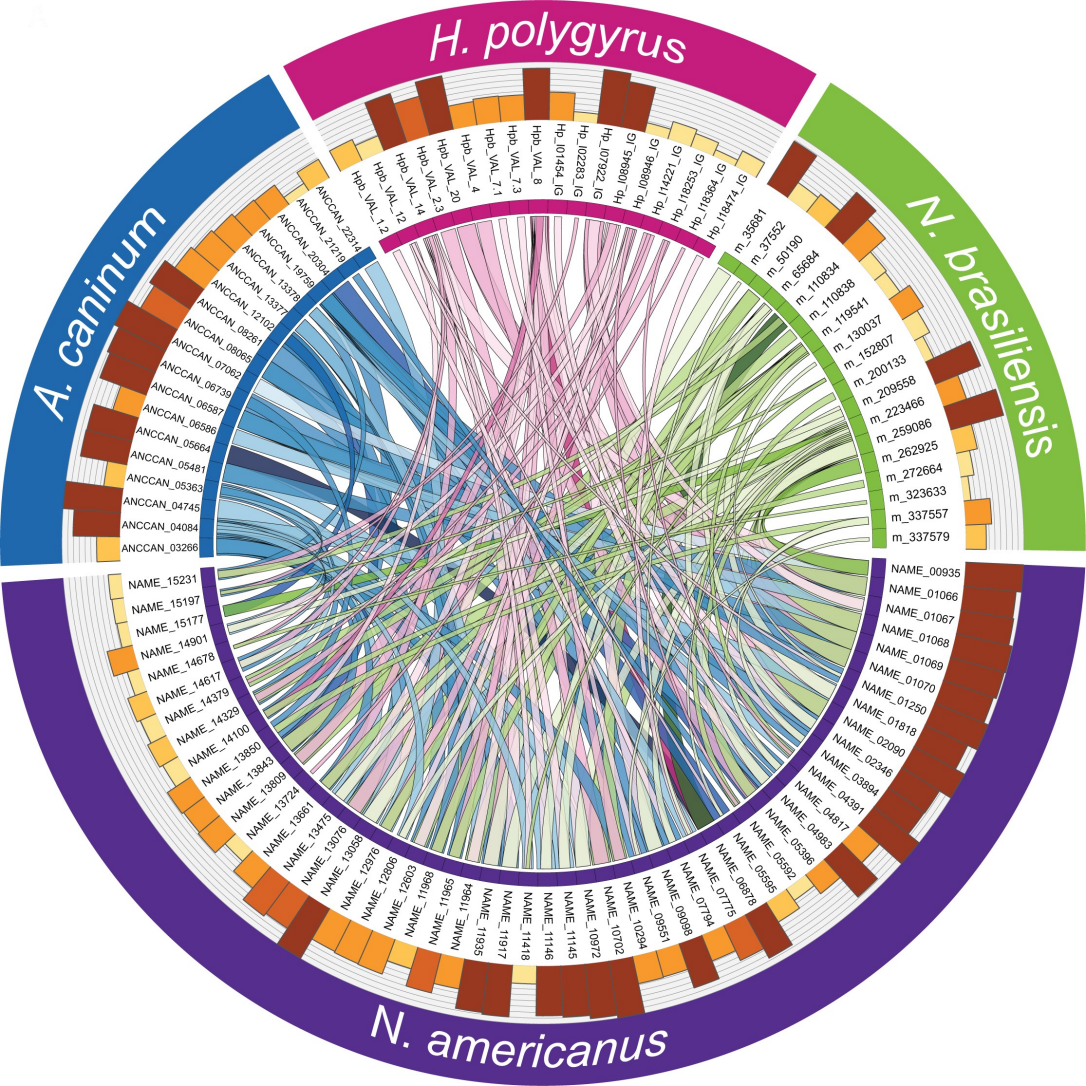
SCP/TAPS proteins in the excretory/secretory products (ES) of *Necator americanus* are most closely related to SCP/TAPS proteins in the ES of *Ancylostoma caninum*. Protein names are displayed in a circle with *N*. *americanus* (purple), *A*. *caninum* (blue), *Heligmosomoides polygyrus* (pink) and *Nippostrongylus brasiliensis* (green). Ribbon thickness is relative to the maximum score obtained in the BLAST search while darker ribbons denote higher sequence percent identity. The corresponding bars provide relative sequence length of each protein.

Since SCP/TAPS proteins and proteases from *N*. *americanus* numerically dominate the ES protein dataset and likely play key roles in infection, migration and parasite establishment, we performed an in-depth analysis of these families of proteins between the human and three model gastrointestinal nematode species. Adult *N*. *americanus* ES SCP/TAPS protein sequences were aligned with homologs from *H*. *polygyrus*, *A*. *caninum* and *N*. *brasiliensis* ES SCP/TAPS protein sequences using BLAST. Sequences were visualized using Circos (Fig 4). SCP/TAPS from *N*. *americanus* are more similar to *A*. *caninum* than *H*. *polygyrus* or *N*. *brasiliensis*, as denoted by thicker, darker ribbons (Fig 4). The sequences, their homologs and the corresponding blast scores are detailed in S10 Table. As with the similarity analysis, NAME_11218 had no significant alignment to any of the compared species.

**Fig 5:**
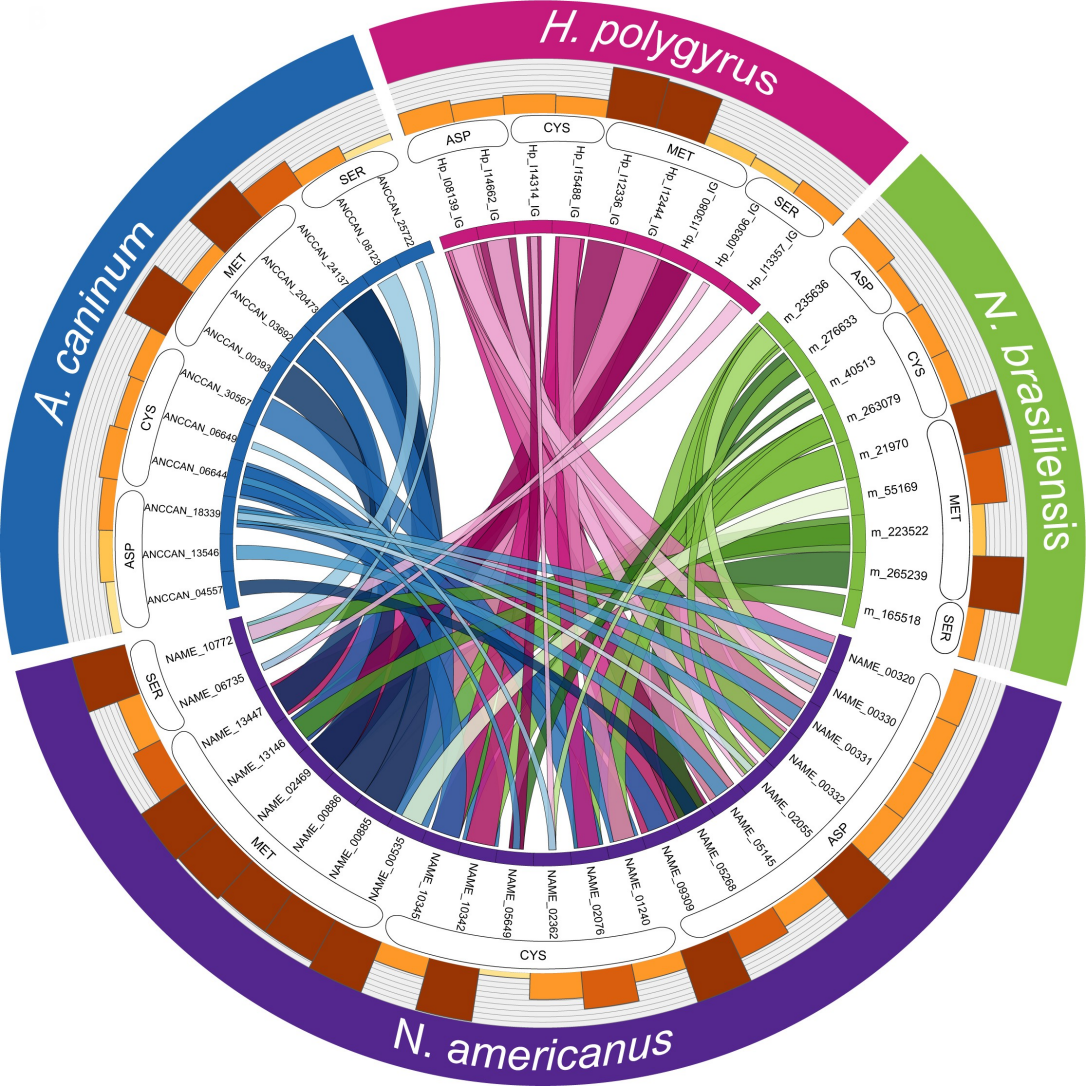
Proteases in the excretory/secretory (ES) products of *Necator americanus* are most closely related to proteases in the ES of *Ancylostoma caninum*. Protein names are displayed in a circle with *N*. *americanus* (purple), *A*. *caninum* (blue), *Heligmosomoides polygyrus* (pink) and *Nippostrongylus brasiliensis* (green). Ribbon thickness is relative to the maximum score obtained in the BLAST search while darker ribbons denote higher sequence percent identity. The corresponding bars provide relative sequence length of each protein. Respective protease mechanistic classes: aspartic (ASP), cysteine (CYS), metallo- (MET) or serine (SER).

A similar analysis was performed for the proteases from each of the aforementioned species (Fig 5). These proteases have been grouped together into their respective mechanistic classes: aspartyl (ASP), cysteine (CYS), metallo (MET) or serine (SER) proteases. In general, all three comparator species had high protease sequence homology to metalloproteases from *N*. *americanus*. Aspartyl protease sequences were more similar between *H*. *polygyrus*, *N*. *brasiliensis* and *N*. *americanus* than sequences from *A*. *caninum*. It is interesting to note that *N*. *americanus* contained more aspartyl proteases than the other nematode species analyzed (*N*. *americanus*– 8; *A*. *caninum*– 4; *H*. *polygyrus*– 6; *N*. *brasiliensis*– 6). Serine proteases had the lowest sequence homology across all of the compared species and protease subclasses.

### Phylogenetic analysis of SCP/TAPS proteins

**Fig 6:**
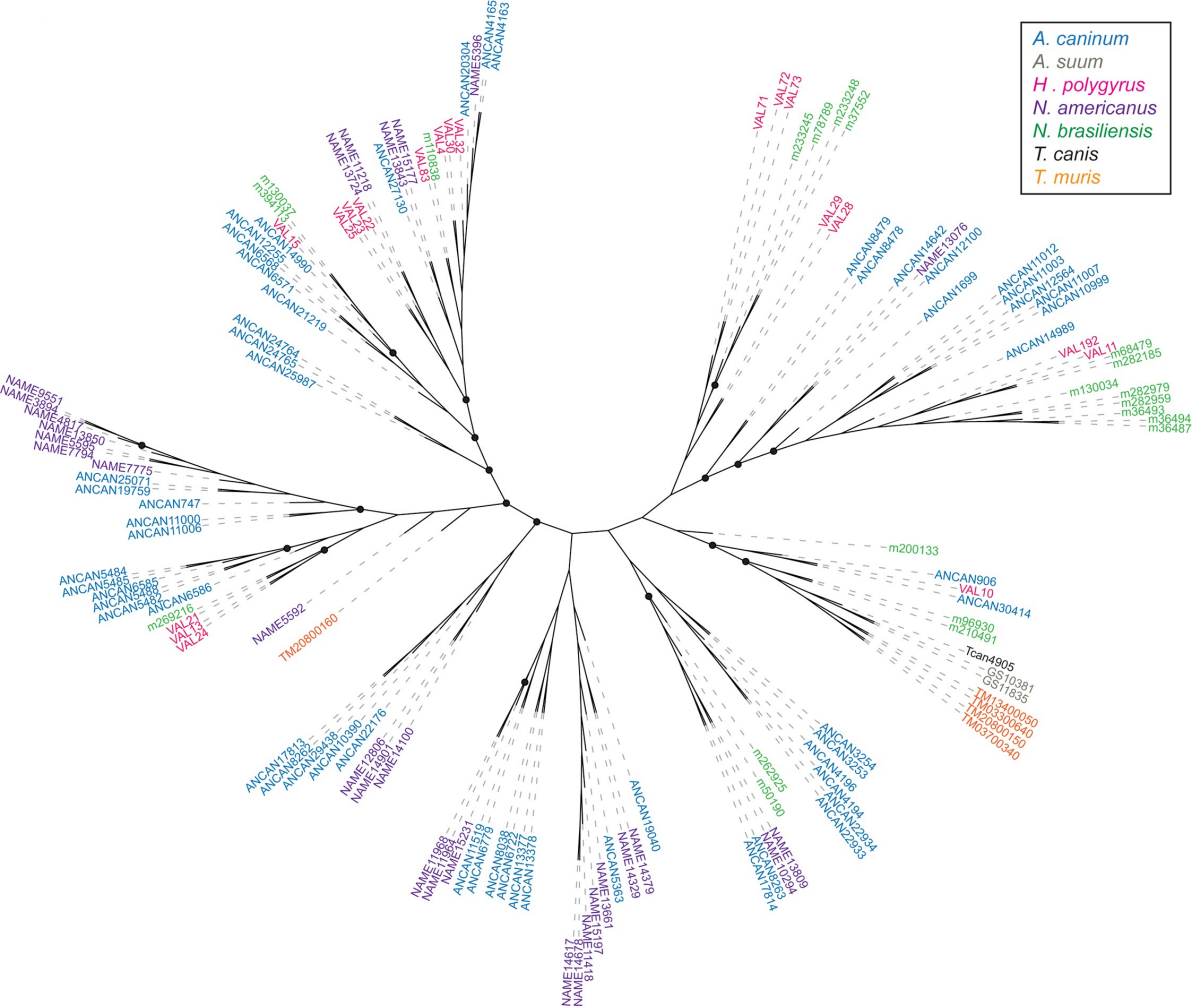
Phylogenetic relationships of single-domain SCP/TAPS proteins determined with MUSCLE alignment software. PhyML was used for a maximum-likelihood phylogenetic analysis with bootstrapping and results were visualized with The Interactive Tree of Life (iTOF) online phylogeny tool. *Necator americanus* sequences are highlighted in purple with comparator species each denoted by a different color (see key). Black solid circles on branches denote bootstrap values with greater than or equal to 70% support.

**Fig 7:**
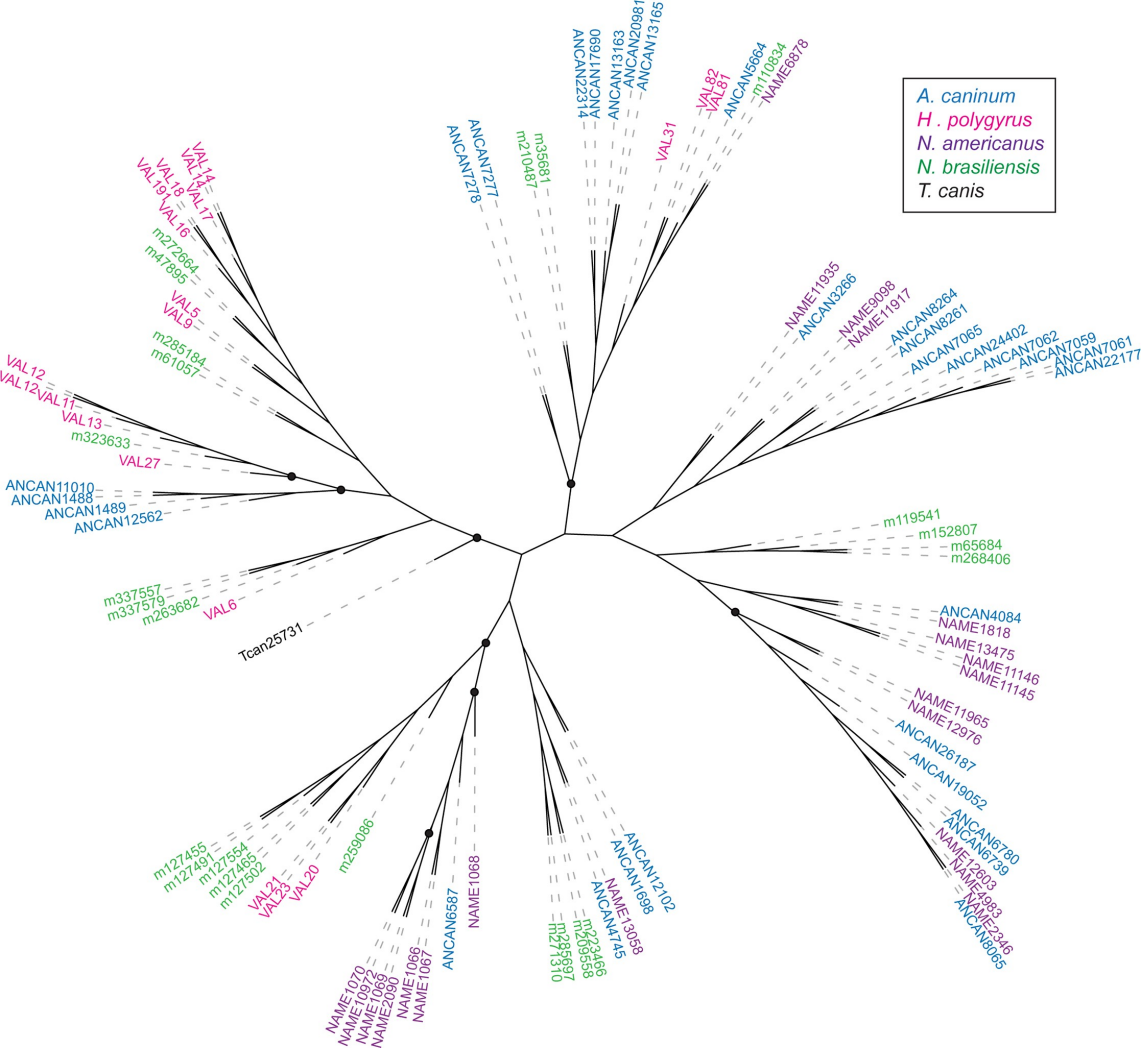
Phylogenetic relationships of double-domain SCP/TAPS proteins determined with MUSCLE alignment software. PhyML was used for a maximum-likelihood phylogenetic analysis with bootstrapping and results were visualized with The Interactive Tree of Life (iTOF) online phylogeny tool. *Necator americanus* sequences are highlighted in purple with comparator species each denoted by a different color (see key). Black solid circles on branches denote bootstrap values with greater than or equal to 70% support.

SCP/TAPS sequences of 6 parasitic nematodes were obtained from published secretomes and compared to *N*. *americanus* SCP/TAPS identified in this study. Sequences were grouped by whether they had a single or double domain and then aligned using MUSCLE and PhyML. From the 7 total species, 232 SCP/TAPS were reported with 134 single-domain proteins and 98 double-domain. For the single SCP/TAPS-domain proteins an unrooted tree was generated. The analysis identified seven main clades (Fig 6). Three of these clades consisted entirely of sequences from *N*. *americanus* and *A*. *caninum*, while sub-clades in 3 of the other main clades followed a similar trend. A majority of *T*. *muris* single-domain sequences formed a sub-clade with *A*. *suum* and *T*. *canis*, grouping together the non-clade V helminths. An unrooted tree was also generated for double domain sequences. The analysis identified five main clades. *N*. *americanus* SCP/TAPS clustered almost exclusively with sequences from *A*. *caninum* again, representing one main clade and several sub-clades (Fig 7). *H*. *polygyrus* and *N*. *brasiliensis* also formed a number of distinct sub-clades. *T*. *canis*, the only non-clade V helminth, was reported to only produce one double-domain SCP/TAPS protein in its ES products which was not closely related to any of the compared sequences. Interestingly, no double-domain SCP/TAPS were reported for *A*. *suum* or *T*. *muris*. Another trend across both single and double domain trees was the diversity of evolution between SCP/TAPS within a single species. For example, the single-domain tree included a sub-clade of 7 *N*. *americanus*-only sequences while other *N*. *americanus* sequences had more similarity to mouse hookworm sequences.

### Diagnostic potential of SCP/TAPS proteins

**Fig 8:**
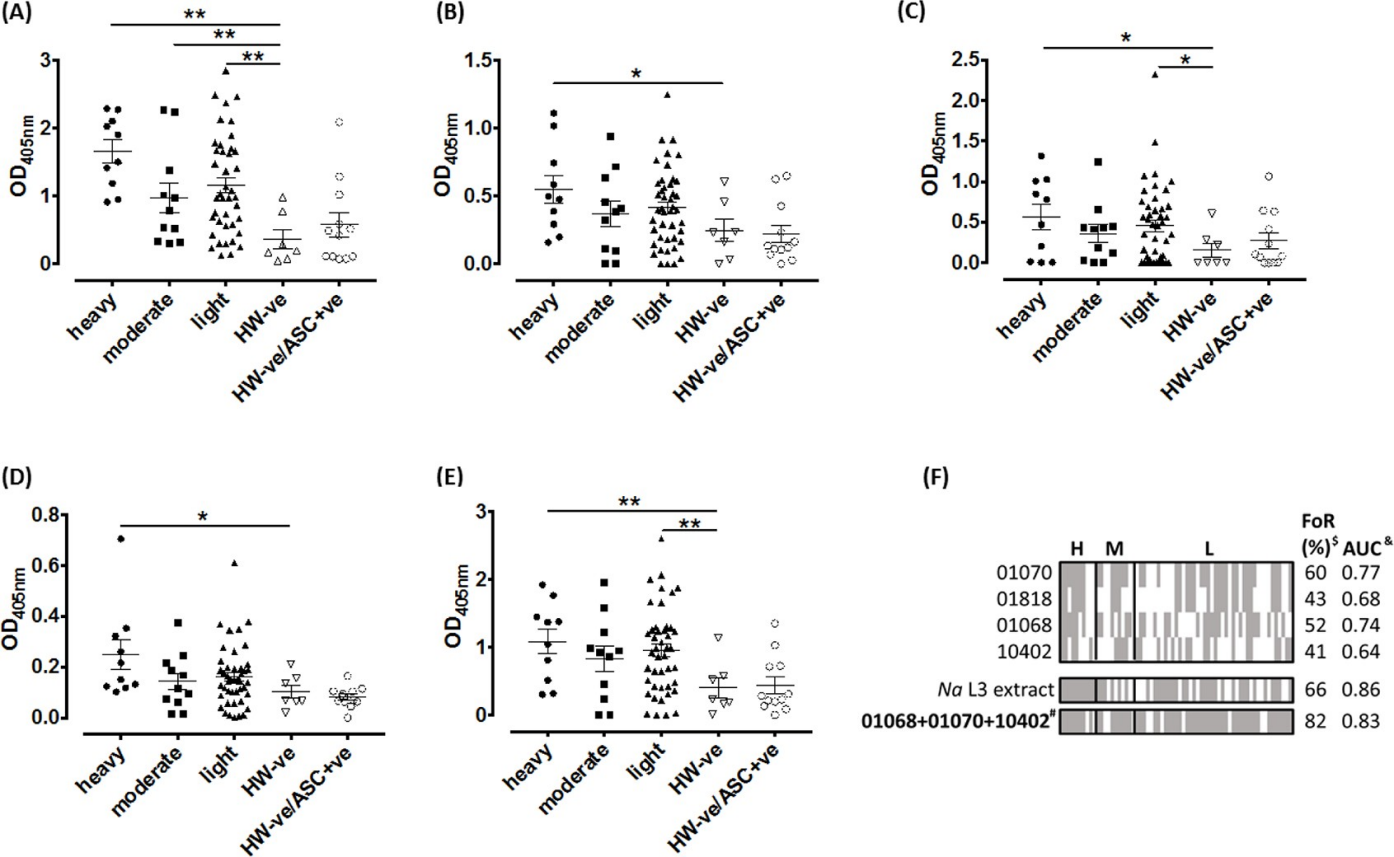
Serological diagnosis of hookworm infection by detection of serum antibodies against hookworm L3 extract and recombinant SCP/TAPS proteins. Scatter plots showing the (A) anti-*N*. *americanus* L3 extract IgG response, (B) anti-01818 IgG response, (C) anti-01068 IgG response, (D) anti-10402 IgG response (E) anti-01070 IgG response. Hookworm-positive subjects were characterized (WHO stratification) as either having a heavy (≥4,000 epg), moderate (2,000–3,999 epg) or light (≤1,999 epg) infection. “HW -ve” = hookworm egg negative subjects from an endemic area, “HW -ve/ASC +ve” = hookworm egg negative, Ascaris egg positive subjects from an endemic area. Differences in responses between groups were analyzed by Student’s *t* test. *P≤0.05, **P≤0.01. (F) Schematic showing frequency of recognition of *N*. *americanus* L3 extract and recombinant SCP/TAPS proteins by hookworm-positive subjects. The recognition cutoff was defined as the average response of the HW -ve group + 1SD. ^#^represents the minimal antigen combination (01070, 01818 and 10402) which gives the highest frequency of recognition. AUC = area under receiver operator curves generated for each antigen and the antigen combination, which determines the positive predictive value of infection.

Using the recombinant *N*. *americanus* SCP/TAPS proteins expressed in this study (and *N*. *americanus* L3 extract as a comparator), we analyzed antibody responses in the serum of *N*. *americanus*-infected individuals (Fig 8A–8E) to each antigen and the AUC generated from the ROC curves (S1 Fig) were used to determine the sensitivity and specificity of each antibody response, and the predictive value of infection (Table 3). Antibodies to all antigens were significantly reactive in subjects with a heavy hookworm infection (≥4,000 epg) compared to hookworm-negative controls. Further anti-01068 and anti-01070 IgG responses were also significant in subjects with a light hookworm load (≤1,999 epg). For the L3 extract and three of the antigens, IgG reactivity in the hookworm-negative/*Ascaris*-positive group was higher (not significantly so) compared to the hookworm-negative group with no *Ascaris* infection. The two *Ascaris* SCP/TAPS proteins share between 16–31% identity with the respective *N*. *americanus* sequences, so overall the similarity is relatively low. The most likely explanation for the small number of *Ascaris*-positive/hookworm-negative individuals is prior or active low intensity infection of these few subjects with hookworm. Hookworm is co-endemic with *Ascaris* in this region of Brazil, and although the subjects were negative for hookworm eggs in the feces, the low sensitivity of the Kato-Katz test means that they might have had low intensity hookworm infection. Of the recombinant antigens tested, the highest positive predictive value of infection (PPV) in hookworm-positive vs hookworm-negative subjects was generated by the antibody response to 01070 (AUC = 0.77). Hookworm-positive subjects with antibody levels against a particular antigen above the reactivity cut-off were recorded as positive for recognition of that antigen and, because the recognition pattern of each antigen among the infected population was different, we investigated whether a combination of antigens would increase the frequency of recognition and/or PPV among hookworm-positive subjects compared to any one antigen alone. A minimum combination of three SCP/TAPS proteins (01070 + 01068 + 10402) increased the FoR to 82% of hookworm-positive subjects, compared to an FoR of 60% and 66% for 01070 and the L3 extract, respectively (Fig 8F and Table 3). Further, this antigen combination gave a PPV of 0.83, which was comparable to 0.86 obtained for the L3 extract.

**Table 3:** Frequency of Recognition and Area Under the Curve values for *N*. *americanus* L3 extract and recombinant SCP/TAPS proteins in the diagnosis of hookworm infection.

Antigen	FoR (%)^2^	AUC^3^
*N*. *americanus* L3 extract	66	0.86
01818	43	0.68
01068	52	0.74
10402	41	0.64
01070	60	0.77
01068 + 01070 + 10402^1^	82	0.83

^1^ the minimal antigen combination (01070, 01818 and 10402) which gives the highest frequency of recognition.

^2^ the recognition cutoff was defined as the average response of the HW -ve group + 1SD.

^3^ area under receiver-operating characteristic curves generated for each antigen and the antigen combination, which determines the positive predictive value of infection.

## Discussion

*Necator americanus* affects more than 400 million people worldwide and is the most important soil transmitted helminth in terms of morbidity [2]. The genome of *N*. *americanus* was sequenced in 2014, providing an important dataset to facilitate efforts to combat hookworm disease; however, the tools available at that time for annotating genes from parasitic helminths were limited. For instance, the number of proteins with a top hit to a ‘hypothetical protein’ present in the original genome annotation was 3,043, corresponding to 15.8% of the total predicted proteins [11]. In addition, inferring gene and protein functions for parasitic nematodes is a major challenge as most species are genetically intractable and databases and algorithms are biased towards (free-living) model nematodes [29]. Proteogenomics is a relatively new approach in which proteomic data is used to improve genome annotation [31, 59], and although it had never been applied to parasitic helminths (until now), its potential utility in this area has been suggested [60]. High-throughput sequencing and gene prediction tools are prone to false-negative and false-positive predictions which can lead to missed genes, false exons or exon boundaries and/or incorrect translational start/stop sites, so knowing the sequences of the proteins expressed by an organism will help to improve gene predictions.

The proteogenomic analysis carried out in this study addresses some of these challenges by improving the characterization of predicted proteins from the annotated *N*. *americanus* genome. Overall, we identified 121 peptides that map to intergenic regions in the first draft genome sequence for *N*. *americanus*. Peptides that map to intergenic regions are highly significant as they can lead to identification of novel protein-coding genes or corrections of pre-existing models [31]. To investigate whether these peptides are likely to be new genes we checked for any potential ORFs where the peptides map. Thirty-two (32) of the peptides identified mapped to alternative ORFs (group 4, Fig 1) than those described in the current gene model. While these peptides map to known coding regions, they highlight out-of-frame ORFs. Of the newly identified peptides, 39 of them mapped to introns. Peptides mapping to introns can lead to identification of novel splice isoforms or amendments in gene structure. Peptides mapping to exon extensions and N-terminal extensions were less abundant, with 10 and 4 peptides respectively. These groups of peptides suggest a possible correction in reading frame or an incorrect start site annotation. All of these ORFs were blasted against a custom database containing three species of *Ancylostoma* and a SMART domain prediction was carried out. Given that 41 of these sequences (S5 Table) had a functional domain, they are likely to correspond to proteins. Further studies are necessary to confirm these functions.

The decrease in gene number seen in this study is in line with other genome annotations. For example, the genome of the parasitic platyhelminth *Schistosoma mansoni* was originally thought to encode 11,809 genes, however further annotation has reduced this number to 10,772 [61]. Given that this is the first re-annotation of the *N*. *americanus* genome since the original draft was published, a substantial decrease in gene number was to be expected, and 15,728 genes is comparable with the predicted gene numbers of other nematode genomes [62]. Despite being a parasitic nematode, *N*. *americanus* has almost 5,000 fewer genes than its free-living relative *C*. *elegans* (https://parasite.wormbase.org/).

Of particular importance in the characterization of parasite-specific genes is the presence of a signal peptide. Of the secreted ES proteins from *N*. *americanus*, 51% were predicted to have a signal peptide; this is also in agreement with ES proteomes from other parasitic nematodes [15, 19, 53]. The presence of extracellular proteins without predicted signal peptides in the ES products of *N*. *americanus* could be due to one of three reasons: (a) the protein is secreted via an alternative pathway, including release of parasite extracellular vesicles [63–66] or non-classical secretory signals; (b) the lack of full-length RNA transcript sequence to confirm gene model accuracy resulted in an error in the predicted sequence (i.e. truncation or ORF shift); (c) the pre-set D-cutoff threshold of 0.33 in SignalP results in false-negative predictions.

Helminth secretomes represent the molecular host-parasite interface [9], and provide useful insights into the biological strategies employed by these parasites to ensure longevity inside their respective hosts [9]. At this interface, ES products have been implicated in numerous roles from initial penetration/invasion and feeding to host immune regulation [9]. Obtaining sufficient ES products to generate the proteome described in this study was time consuming due to the difficulty in culturing sufficient quantities of parasites in hamsters. For this reason, human infection with *N*. *americanus* is frequently modelled using other hookworms and related nematodes that survive in rodents or larger animals, including *Ancylostoma sp*., *H*. *polygyrus* and *N*. *brasiliensis*. Adult stage *N*. *americanus* parasites obtained from experimentally infected hamsters are smaller and less fecund than those from human hosts [67]. These phenotypic differences may be associated with differences in transcriptional patterns. To date there have been no studies directly comparing the genomes or secretomes of *N*. *americanus* maintained in hamsters and those obtained directly from human infections. Such data would enhance the validity of the data obtained in this study. The protein family analysis of *N*. *americanus* ES products revealed a diverse number of known and unknown domains (391 domains total), which attests to the many biological functions of ES proteins as well as to the lack of information on these proteomes. The ES products of the three comparator species used here had similar protein family profiles with 458 (*A*. *caninum*), 434 (*H*. *polygyrus*) and 628 (*N*. *brasiliensis*) unique domains present. To assess the overall usefulness of these models, a similarity analysis was carried out on their ES products. The majority of the *N*. *americanus* ES proteins (173/198), including 51/54 SCP/TAPS proteins, had homologs in the ES products of all three comparators, highlighting the relevance and usefulness of all three models. A total of 25 proteins did not have similarity to ES proteins from the other 3 nematodes analyzed. Since *A*. *caninum*, *H*. *polygyrus* and *N*. *brasiliensis* are animal parasites, these 25 proteins might have evolved to such an extent that they target human-specific pathways. Two unique proteins of interest are the aspartyl protease NAME_01848 (PF00026) and the zinc metalloprotease NAME_05081 (PF01546). Metalloproteases and aspartyl proteases play crucial roles in host tissue penetration and parasite feeding, and as such, proteolytic enzymes from helminths may be of particular interest as vaccine and/or drug targets [68, 69].

The most abundantly represented protein family (54/198; 27%) in *N*. *americanus* ES products was proteins containing a single or double SCP/TAPS domain (PF00188). This finding aligns with previous work that highlighted the abundance of this protein family in clade V nematode ES products in particular [15, 70]. For instance, a total of 45, 90 and 25 SCP/TAPS proteins were found in the ES products from *N*. *brasiliensis* (45/313; 14%), *A*. *caninum* (90/315; 29%) and *H*. *polygyrus* respectively (25/374; 12%). Interestingly, this family of proteins is also abundant in the extracellular vesicles secreted by different nematodes [65, 66]. Given that SCP/TAPS proteins from *N*. *brasiliensis* are more abundantly secreted by the adult developmental stage [19], many of these proteins are likely coordinating specific roles in the gastrointestinal tract of the host. In fact, it has also been shown that SCP/TAPS are overrepresented at the transcript level in *N*. *americanus* adult worms [11]. While relatively little functional information is available for SCP/TAPS proteins, neutrophil inhibitory factor (NIF), an SCP/TAPS protein in the ES of *A*. *caninum*, was reported to abrogate neutrophil adhesion to the endothelium [71]. However, a *N*. *americanus* homolog of this protein was not detected in the current study, despite the presence of a NIF-encoding gene in the draft genome [11]. SCP/TAPS proteins are thought to play numerous and diverse roles at the host-parasite interface, from defense mechanisms, normal body formation and lifespan [55]. SCP/TAPS from both *A*. *caninum* and *N*. *americanus* have been used in vaccine studies in mice, dogs and humans and displayed partial efficacy in animals [72, 73].

The diverse nature of *N*. *americanus* SCP/TAPS sequences is evidenced by their phylogenetic relationships (Figs 4–7) and varying degrees of sequence homology to SCP/TAPS proteins from *A*. *caninum*, *H*. *polygyrus* or *N*. *brasiliensis*. These SCP/TAPS proteins should be further explored to understand their roles in *N*. *americanus*-human host interactions. Given the limited availability of information regarding the function of helminth SCP/TAPS proteins in general, it was unsurprising that GO analyses revealed no known molecular function or biological process for 32/54 of the SCP/TAPS from *N*. *americanus* ES products, and more studies should be performed to characterize the properties of this intriguing family of proteins.

Despite the lack of functional information on the SCP/TAPS proteins in parasitic helminths, of the species we compared in this study, *N*. *americanus* proteins were generally most similar to those from *A*. *caninum*, which could simply be a reflection of the phylogenetic similarity between the two species. The trees generated in this study highlight strong clade-specific similarities between SCP/TAPS in the ES products of the compared species. In support of this, the vast majority of the SCP/TAPS proteins came from the four clade V species, while *A*. *suum*, *T*. *muris* and *T*. *canis* had only 2, 5 and 2 SCP/TAPS proteins respectively. The clustering of *A*. *caninum* with *N*. *americanus* and *H*. *polygyrus* with *N*. *brasiliensis* supports the notion of host-specific roles for SCP/TAPS. Another trend across both single and double domain trees was the diversity of evolution between SCP/TAPS within a single species. For example, the single-domain tree included a sub-clade of 7 *N*. *americanus*-only sequences indicating an important human-specific role for this evolutionary cluster. This compares well with a number of other sub-clades which included proteins from the four predominant species. These SCP/TAPS proteins are more likely to share a common function, potentially in host-infection or parasite development.

The phylogenetic analysis strongly attests to the preferred use of *A*. *caninum* for studying hookworm molecular biology and ES products in general [74]. This finding is reinforced by the Circos plot of SCP/TAPS (Fig 4). Not only was there a greater number of SCP/TAPS homologs in the ES products of *A*. *caninum* but these proteins also had relatively higher blast scores (denoted by link ribbon thickness) and higher percent identity scores (denoted by ribbon darkness). This type of analysis can prove useful since it also reveals which species to consider for investigating a specific *Necator* SCP/TAPS protein. For example, NAME_13850 has significant sequence homology to a SCP/TAPS protein from each of the compared species; however, the species with the highest sequence homology is *A*. *caninun* (ANCCAN_19759), making this protein the most relevant to study as a model for NAME_13850. Prior to updating the genome, all three species of nematode used in this comparison had longer average gene sequence lengths than the *N*. *americanus* SCP/TAPS sequences. In the previous annotation, the average *N*. *americanus* SCP/TAPS sequence length was 244 predicted amino acids, compared with 355 residues in the updated genome. This was likely due to some of the sequences being truncated, yielding similar results to the published *H*. *polygyrus* proteome [53].

The Circos plot representing *N*. *americanus* proteases and homologous proteins from the three comparator species (Fig 5) provides insight into the high degree of sequence similarity. Unlike the SCP/TAPS Circos analysis, all the *N*. *americanus* ES proteases had homologs with high similarity in the compared species (average 50% identity between *N*. *americanus* and comparator species protease). This finding supports the notion of using any one of these three parasites to study *N*. *americanus* proteases in general. We identified all four major mechanistic classes of protease in the ES products of *N*. *americanus*. As the adult stage of *N*. *americanus* feeds on blood, the high abundance of aspartyl proteases was expected [25, 75]. Yet when compared to other species, the human hookworm ES products included more of these proteases. Aspartyl proteases play a fundamental role in the digestion of host hemoglobin and have also been implicated in skin penetration, feeding, and host tissue degradation [76, 77]. The finding that *N*. *americanus* has more of this mechanistic class of proteases than the other nematodes assessed here is likely due to split gene models and/or may be a true gene family expansion. Due to the vital role that aspartyl proteases play in parasite feeding, *Na*-APR-1 an *N*. *americanus* aspartyl protease, was targeted as a vaccine candidate [78]. While we were unable to detect *Na*-APR-1 in the current ES proteome—probably because it is anchored to the gut epithelium [79] - 9 other aspartyl proteases were detected which could potentially be targeted as novel vaccine candidates.

Cysteine proteases, particularly the group belonging to the papain superfamily, are common in nematodes [80]. They have been specifically described for their proteolytic activity against hemoglobin, antibodies and fibrinogen in the *N*. *americanus* lifecycle [81]. Similarly, another study highlighted the importance of four cysteine proteases that were upregulated in the *N*. *americanus* transition from free-living larvae to blood-feeding adult worm, indicating that these proteins are likely to be important for nutrient acquisition [82]. Metalloproteases—particularly the astacins—identified in this study are most likely important for larval and adult migration and invasion through human host tissue [83]. In support of this, astacin metalloproteases were found to be upregulated in *N*. *brasiliensis* larvae when compared with adult stage parasites [19]. Interestingly, *N*. *americanus* metalloproteases were reported to inhibit eosinophil recruitment through the cleavage of eotaxin, a potent eosinophil chemoattractant [23]. The least abundant family of proteases in the adult *N*. *americanus* ES products were the serine proteases. Relatively little is known about these proteases from *N*. *americanus* specifically; however, a serine protease from the whipworm *T*. *muris* is involved in degradation of the mucus barrier to facilitate feeding [84]. Due to the importance of these various proteases in parasite feeding, infection, migration and defense, they represent potential targets for chemotherapies and vaccines to limit infection [85].

In a demonstration of the potential translatability of the “omics” data generated herein, we recombinantly expressed four of the SCP/TAPS proteins identified in this study and assessed their potential to diagnose hookworm infection in an endemic area in Brazil by measuring serum IgG levels to each antigen in individuals who were positive for the presence of parasite eggs in the feces, the current gold standard for diagnosis. When the diagnostic performance of the antigens was assessed individually, each molecule was found to be an accurate predictor of infection as each cognate antibody response generated an AUC value above 0.5, where an AUC of 1.0 is a perfectly accurate test [86]. Regarding individual recombinant antigens, 01070 displayed the highest FoR (60%) among the infected population but this value was increased by over 20% when the FOR of a combination of antigens (01070, 01068 and 10402) was calculated, due to the varying recognition patterns of each antigen among hookworm-positive individuals. Further, this antigen combination had an FoR that exceeded, and an AUC that was comparable to, that of the L3 extract, which could be considered the gold standard for serodiagnosis of hookworm disease, given the widespread use of parasite extracts as markers of helminth infection. While the PPV of infection of the L3 extract slightly exceeded that of the antigen combination (understandable given the complexity of the preparation, and so it’s ability to capture the breadth of the anti-hookworm immune response) the advantages of a defined, recombinant antigen preparation lies in its reproducibility and sustainably as a resource, compared to, especially in the case of human hookworm, the limitations of generating sufficient reagent for use.

In the current study, we have provided the first proteogenomic analysis of a helminth parasite, resulting in a more accurate genome annotation. While the above annotations are valuable additions to the current genome, further improvement requires access to substantially more parasitic material including adult stage parasites which are difficult to obtain. Furthermore, we have carried out the first proteomic analysis of the ES products of the human hookworm *N*. *americanus*. The results presented herein offer significant insight into the validity of these model species while also highlighting differences between these important parasites.

## Supporting information

S1 Table SCP/TAPS protein sequences used for phylogenetic comparison of six parasitic secretomes. (ZIP)

S2 Table Proteogenomics data showing newly identified peptides translated protein sequences with their respective peptides, six-frame translated genome ID and ORF length. (XLSX)

S3 Table Analysis of peptides mapping to intergenic regions for ORF and methionine. (XLSX)

S4 Table ORFs blasted against a custom database containing three species of *Ancylostoma*. (XLSX)

S5 Table SMART domain prediction of ORFs identified in proteogenomic analysis. (XLSX)

S6 Table Identified contaminant and host proteins (*M*. *auratus)*. (XLSX)

S7 Table Analysis of LC-MS/MS results using Mascot, X!Tandem and Comet searches. (XLSX)

S8 Table Pfam domains of each *N*. *americanus* sequence detected using HMMER software. (XLSX)

S9 Table Gene ontology annotation of *N*. *americanus* ES proteins using Blast2GO. (XLSX)

S10 Table Multiple species blast used for Circos analysis of SCP/TAPS. (XLSX)

S1 Fig ROC curves of *N*. *americanus*-infected serum to each recombinantly produced antigen. (PNG)
