# Supplementary figures and images for: Comprehensive analysis of the secreted proteome of adult Necator americanus hookworms
Source: PLoS Negl Trop Dis. 2020 May 26;14(5):e0008237. doi: 10.1371/journal.pntd.0008237 (PMC7274458; doi:10.1371/journal.pntd.0008237)

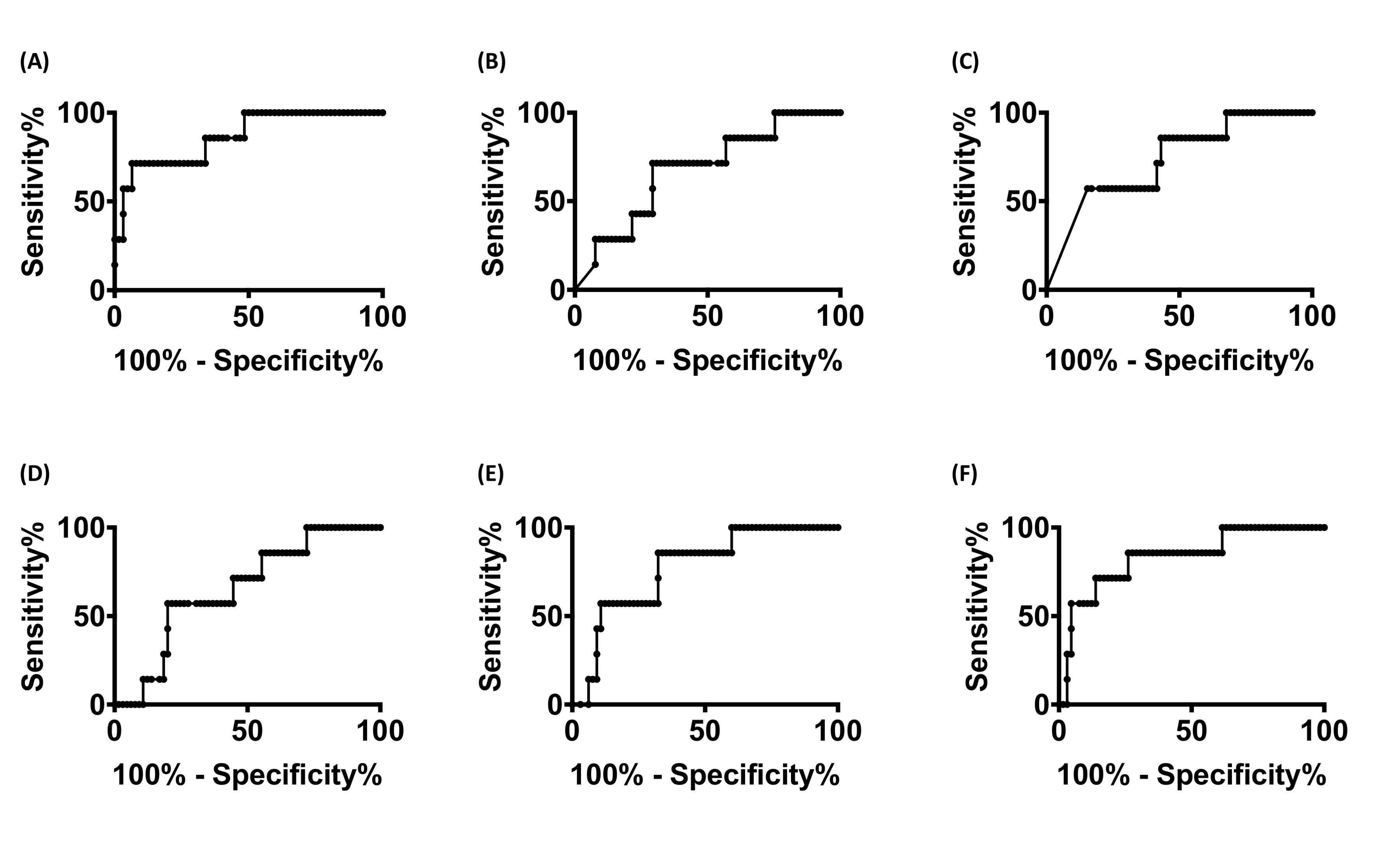

Supplement: S1 Fig — (PNG) [file pntd.0008237.s011.png]
